# Cross walk between consensus recommendations and new NCATS PAR-21-293 requirements for D&I in CTSA hubs

**DOI:** 10.1017/cts.2022.4

**Published:** 2022-01-24

**Authors:** Jane E. Mahoney, Kathleen R. Stevens, Tara Mehta

**Affiliations:** 1 Geriatrics and Gerontology, School of Medicine and Public Health, University of Wisconsin-Madison, Madison, Wisconsin, USA; 2 School of Nursing and Institute for Integration of Medicine & Science, University of Texas Health Science Center San Antonio, San Antonio, Texas, USA; 3 Department of Psychiatry, College of Medicine, University of Illinois Chicago, Chicago, Illinois, USA

The National Center for Advancing Translational Sciences (NCATS) has awarded Clinical and Translational Science Awards (CTSA) to more than 50 medical centers in the United States to accelerate the pace of translational research to improve human health. NCATS recently issued a new Funding Opportunity Announcement, PAR-21-293, that will apply to all new and renewal proposals for CTSA hubs beginning in 2022 [1]. PAR-21-293 newly requires each hub to have foundational dissemination and implementation (D&I) capabilities and activities to ensure that translational research results in health impact. In a 2021 article [2], members of the D&I working group of the NCATS consortium shared a vision of how CTSA hubs could integrate D&I sciences into each hub’s methods and processes, workforce development, and evaluation components in order to speed the translation of research into practice. This editorial crosswalks recommendations from the 2021 publication with the new NCATS requirement and describes strategies that CTSA hubs can use to enhance the integration of D&I sciences into the required elements of their application, in line with PAR-21-293.

CTSA Program hubs at 50 medical centers in the United States provide innovative resources, training, and mentorship to investigators to improve the efficiency, quality, and impact of translational research to improve the health of individuals and communities. PAR 21-293 highlights the imperative for CTSA hubs to translate research findings into healthcare practice and community health settings, and specifically requires each hub to develop D&I capabilities and utilize D&I activities to ensure that translational research results in health impact. PAR 21-293 organizes CTSA hub proposals into operational elements. Element B, the Strategic Management Element, includes all activities related to management of the hub to ensure the hub achieves its objectives. PAR 21-293 states that, as part of the Strategic Management Element, “Each CTSA hub is required to engage in D&I activities to support innovative approaches to identifying, understanding, and developing strategies for overcoming barriers to the adoption, adaptation, integration, scale-up and sustainability of evidence-based interventions, tools, policies, and guidelines” with the goal to deliver the benefits of translational science to all. While the PAR provides opportunities for incorporation of D&I sciences within other elements of the hub (e.g., training and outreach, core resources and services), the responsibility for management of D&I resources is centralized within the strategic management element.

PAR-21-293 represents an important and substantive change in CTSA awards. Previously, D&I resources have not been a required component of CTSA hubs. Some CTSA hubs provide D&I scientific resources, most typically as part of community engagement cores [3]. PAR 21-293 broadens the scope of D&I beyond its original siloed home in the community engagement core and urges integration of D&I within all components of the CTSA hub. The requirement for D&I activities in PAR 21-293, and the location of these activities within the strategic management element, clearly emphasizes the imperative to bridge the research to practice gap. Centralization of D&I resources in the strategic management element should help ensure that all elements of the CTSA hub, from training and outreach, to core resources and services, leverage D&I sciences. Increased utilization of D&I sciences within all elements of CTSA hubs should speed translational research and increase its relevance for clinical partners, stakeholders, and communities [1,3]. PAR 21-293 further requires that hubs disseminate their advancements in clinical and translational science (CTS) to other hubs. The responsibility for dissemination of CTS innovations also rests within the strategic management element and it is expected that the hub will utilize D&I activities to do so.

In this editorial, we highlight two resources produced by members of the NCATS consortium D&I workgroup that can support applicants to address the new requirements in the current PAR. In a 2019 publication [4], members of our NCATS consortium D&I working group proposed a framework that illustrated how D&I sciences can benefit translational endeavors within and between all stages of the translational research spectrum (basic research, preclinical research, clinical research, clinical implementation, public health), ultimately speeding the translation of research towards practice adoption and improved health outcomes and health equity. In a 2021 article [1], members of our D&I working group shared a vision of how CTSA hubs could integrate D&I sciences into their methods and processes, workforce development, and evaluation components. We recommended approaches, and provided examples of promising practices, that our group of experts agreed were useful to help embed D&I sciences into CTSA processes and resources to support translational research. Finally, we emphasized that D&I sciences should be applied to support research across all stages of the translational research spectrum and into practice.

In order to provide initial guidance to CTSA hubs as they respond to the new PAR, Table 1 summarizes our 2021 consensus recommendations for CTSA hubs to integrate D&I sciences into methods and processes, workforce development, and evaluation and crosswalks these consensus recommendations with the NCATS requirements in PAR 21-293. Table 1 additionally shows how our consensus recommendations can be utilized to fulfill the requirement in PAR 21-293 for innovation in CTS “that will be catalytic to translational efficiency and the development and delivery of interventions that improve the health of individuals and communities.” PAR 21-293 recommends areas for potential innovation within each of the elements. In addition, Elements D (Translational Science Pilots) and E (Clinical and Translational Science Research Program) afford opportunities to design, test, and disseminate innovative methodologies, programs and practices that harness D&I sciences to optimize translational research and the adoption and implementation of research into practice.

**Table 1.** Recommendations to enhance integration of D&I sciences within CTSAs [1] and corresponding requirements and opportunities in the new NCATS PAR-21-293 [2]

Recommendations	PAR-21-293 requirements and opportunities for innovation
Methods and processes	
Secure University support for D&I as part of CTSA hubs (e.g., pilot funding for D&I research)	
Incorporate D&I scientific frameworks into CTSAs’ resources for recruitment to inform methods to overcome barriers to recruitment	Module D1, Core Resources and Services: includes recruitment resources *Potential for innovation*: Clinical study recruitment improvements and tools
Integrate D&I science into stakeholder engagement and team science resources, to ensure that, beginning with earliest stages of research, research-generated solutions are useful, usable, desirable, and designed to maximize adoption and spread in practice.	Module C, Outreach: “Stakeholders may be engaged in identifying, developing, demonstrating, disseminating, or adhering to an intervention, method, or tool.” *Potential for innovation:* Science of community and stakeholder engagement
Incorporate D&I scientific methods into resources for study design, to maximize external validity and ensure feasibility, usability, and effectiveness of results in “real world” setting through use of pragmatic and hybrid design methods	Module D, Core Resources and Services: “address the many stages of CTS research, including planning, conduct, analyses, implementation and dissemination.” *Potential for innovation*: Contemporary clinical trial designs; clinical outcome criteria (e.g., patient-reported outcomes); shortening time of intervention adoption
Support D&I activities necessary for the successful translation of innovations into practice, particularly those with high impact but low market potential.	Module B, Strategic Management Element: “Each CTSA hub is required to engage in Dissemination and Implementation (D&I) activities to support innovative approaches to identifying, understanding, and developing strategies for overcoming barriers to the adoption, adaptation, integration, scale-up and sustainability of evidence-based interventions, tools, policies, and guidelines.” “Each hub is expected to have a plan for building and disseminating an evidence-base for each CTS science endeavor, including Pilot projects (Element D), and CTS Research program (Element E).” *Potential for innovation*: Methods to promote adoption and integration of evidence-based practices, interventions, and policies into health care and public health settings to improve impact; processes and programs to support and advance translation; shortening time of intervention adoption
Create D&I infrastructure, such as a D&I core, to: Administer D&I research pilots Collaborate and ensure integration of D&I with other CTSA resources Engage D&I consultants to support researchers across translational research spectrum Provide services for translating and packaging innovations for broad scale-up Support stakeholder-engaged networks to facilitation spread and adoption of innovations Facilitate cross-CTSA collaboration to advance D&I science and share resources	Module B, Strategic Management: “Each CTSA hub is required to engage in Dissemination and Implementation (D&I) activities to support innovative approaches to identifying, understanding, and developing strategies for overcoming barriers to the adoption, adaptation, integration, scale-up and sustainability of evidence-based interventions, tools, policies, and guidelines.” “Each hub is expected to have a plan for building and disseminating an evidence-base for each CTS science endeavor, including Pilot projects (Element D), and CTS Research program (Element E).” *Potential for innovation*: Processes and programs to support and advance translation; shortening time to adoption; methods to better measure impact on health
Workforce development	
Enhance existing training programs with training in D&I competencies and principles, including trainees at all levels (researchers, K and T level trainees, doctoral and post-doctoral students, masters students, tailoring content to researchers’ needs across the translational research spectrum, and focusing on application of D&I principles to trainee’s research area.	Module C, Training: “Professional development must be directed towards members of CTS research teams and other clinical professionals such as clinical investigators, co-investigators, clinical researchers, research nurses, pharmacists, administrators, coordinators, consultants, data managers, quality assurance managers, regulatory affairs managers or educators in clinical trial management.”* *Potential for innovation*: Identify best practices in translational research and workforce education *See also: K12 Clinical Scientist Institutional Career Development Program Award (NOT-TR-21-030).
Provide D&I expertise to early- and late-stage translational research teams through any of: Consultations with D&I scientists Integrating D&I scientists on research teams Regularly scheduled peer learning opportunities (eg D&I working-groups, works-in-progress meetings, learning collaboratives)	Module B, Strategic Management, and Module C, Training* *Potential for innovation*: Identify best practices in translational research and workforce education; develop innovative or improved approaches to the dissemination of educational content (e.g., use of interactive online platforms to optimize the end-user’s experience) *See also: Training and K12 Clinical Scientist Institutional Career Development Program Award (NOT-TR-21-030)
Utilize nationally available training and professional development opportunities in D&I (e.g., training programs; conferences, workshops, and short courses; recorded online training videos)	Module C, Training. FOA encourages applicants to adopt translational science innovations from other hubs where applicable.
Train D&I partners (e.g., quality improvement specialists, practice change facilitators, clinicians, community stakeholders engaged in implementation) in D&I competencies to support research-practice partnerships, and maximize adoption, implementation, reach, and effectiveness of research innovations into practice	Module C, Outreach, Community and Stakeholder Engagement Research Module: “Progression from scientific discoveries to demonstrated improvements in public health requires translational research teams of scientists, clinicians, research participants, patients, and other stakeholders with a wide range of expertise and perspectives.” *Potential for innovation*: Processes and programs to support and advance translation: education/training
Evaluation	
Track acquisition of D&I competencies and skills among faculty from across the translational research spectrum, and their application into research practice.	Module B, Strategic Management: “Strategic management also includes the ongoing planning, monitoring, analysis, and assessment of all that is necessary for an organization to meet its goals and objectives.” *Potential for innovation*: Methods to better measure impact on health
Include D&I scientists in External Advisory Committees for CTSAs, to assist with conceptualizing methods and measures to evaluate D&I capacity, integration into CTSA structures, and impact.	Module B, Strategic Management: “The expertise of the members [of internal and external advisory committees] must be broad and include those with a range of important perspectives such as community representatives, patients, community-based clinicians, health systems representatives, experts in informatics, and industry.” *Potential for innovation*: Methods to better measure impact on health
Track the impact of CTSA-supported research, through use of the Translational Science Benefits Model [5] or other tools, and document and measure the extent to which research-based innovations are scaled up into practice.	Module B, Strategic Management: “Each CTSA hub is required to engage in Dissemination and Implementation (D&I) activities to support innovative approaches to identifying, understanding, and developing strategies for overcoming barriers to the adoption, adaptation, integration, scale-up and sustainability of evidence-based interventions, tools, policies, and guidelines.” *Potential for innovation*: Methods to better measure impact on health

CTSA, Clinical and Translational Science Award; CTS, Clinical and translational science; D&I, Dissemination and Implementation; FOA, Funding Opportunity Announcement.

In addition to the recommended approaches shown in Table 1, our 2021 publication listed 12 areas where further research is needed to understand how to optimize translational research and the adoption and implementation of research into practice. We provided five recommendations for further research on novel ways to incorporate D&I sciences in methods and processes to support translational science; four recommendations for further research to identify core D&I competencies needed by different sectors of the workforce; and three recommendations for research on how best to evaluate the processes and outcomes of integration of D&I sciences into translational research, track the scale-up of innovations into practice, and evaluate the ultimate impact of translational research endeavors on improving health and reducing disparities. Our recommendations are highly congruent with the areas for translational science innovation that are highlighted in PAR 21-293. Elements D and E of PAR 21-293 provide opportunities for further research along the lines of these recommendations.

Ultimately, PAR 21-293 promotes the stronger incorporation of D&I sciences into the work of CTSA hubs to enhance the translation of research into practice to improve health and reduce disparities. The publications from members of the D&I working group of the CTSA consortium [1,3] provide a starting point for CTSA hubs as they respond to the new requirements of PAR 21-293 and suggest opportunities to design, study, and disseminate translational science innovations. We hope our recommended approaches and the corresponding examples of promising practices will help CTSA hubs meet the new NCATS requirements.

The D&I working group of the CTSA consortium continues to identify and disseminate best practices and welcomes new CTSA members to join.
